# Activatable fluorescent probes for imaging and diagnosis of rheumatoid arthritis

**DOI:** 10.1186/s40779-023-00467-7

**Published:** 2023-07-14

**Authors:** Pan Luo, Fu-Qiang Gao, Wei Sun, Jun-You Li, Cheng Wang, Qing-Yu Zhang, Zhi-Zhuo Li, Peng Xu

**Affiliations:** 1 Department of Joint Surgery, Honghui Hospital, Xi’an Jiaotong University, Xi’an, 710054 China; 2 Department of Orthopedics, China-Japan Friendship Hospital, Beijing, 100029 China; 3 Department of Orthopaedic Surgery of the Perelman School of Medicine, University of Pennsylvania, Philadelphia, PA 19104 USA; 4 School of Mechanical Engineering, Sungkyunkwan University, Suwon, 16419 South Korea; 5 Department of Orthopaedic Surgery, Peking University Third Hospital, Peking University, Beijing, 100191 China; 6 Department of Orthopedics, Shandong Provincial Hospital Affiliated to Shandong First Medical University, Jinan, 250021 China; 7 State Key Laboratory of Pharmaceutical Biotechnology, Division of Sports Medicine and Adult Reconstructive Surgery, Department of Orthopedic Surgery, Nanjing Drum Tower Hospital, the Affiliated Hospital of Nanjing University Medical School, Nanjing, 210008 China

**Keywords:** Rheumatoid arthritis, Fluorescent probe, Imaging, Diagnosis, Biomarker

## Abstract

Rheumatoid arthritis (RA) is a systemic autoimmune disease that is primarily manifested as synovitis and polyarticular opacity and typically leads to serious joint damage and irreversible disability, thus adversely affecting locomotion ability and life quality. Consequently, good prognosis heavily relies on the early diagnosis and effective therapeutic monitoring of RA. Activatable fluorescent probes play vital roles in the detection and imaging of biomarkers for disease diagnosis and in vivo imaging. Herein, we review the fluorescent probes developed for the detection and imaging of RA biomarkers, namely reactive oxygen/nitrogen species (hypochlorous acid, peroxynitrite, hydroxyl radical, nitroxyl), pH, and cysteine, and address the related challenges and prospects to inspire the design of novel fluorescent probes and the improvement of their performance in RA studies.

## Background

Rheumatoid arthritis (RA), a systemic autoimmune inflammatory disease associated with many complex factors [1, 2], is manifested as joint swelling, severe pain, stiffness, synovitis, and cartilage damage [3–6], and affects 0.5–1.0% of the global population [7]. The adverse effects of RA include physical and mental problems due to long-term pain as well as irreversible joint damage or even disability (in severe cases) [8–10]. Currently, the drugs used to treat RA or relieve the associated pain are ineffective in approximately one-third of patients [11, 12], and RA incidence alleviation or reduction remains a key concern [13, 14]. Therefore, early diagnosis is necessary to ensure timely RA treatment and thus effectively retard disease progression, avoid joint necrosis or disability, and ensure a normal life.

One of the earliest symptoms of RA onset is synovial inflammation; however, the early pathogenesis of RA remains unknown [15, 16]. Although medical imaging is highly important for clinical practice and has become a major tool for RA diagnosis and treatment [17], conventional imaging methods and radiographic examinations primarily depend on the presence of relatively advanced features and are insufficiently sensitive for early diagnosis [18, 19]. Magnetic resonance imaging (MRI), which is more accurate than conventional X-ray imaging, can provide clear anatomical information regarding inflammation within the joints and thus help to effectively predict early-stage disease progression [20, 21], but it is more expensive than conventional screening and is not available on a large scale [22]. In addition, purely anatomical imaging does not always detect abnormal changes such as cellular, molecular, or physiological alterations in the early stages of RA pathogenesis [23]. Unlike conventional non-invasive imaging modalities, molecular imaging is suitable for early RA diagnosis [24], primarily relying on non-invasive light-matter interactions in cells or tissues and analysing the corresponding optical signals [25–29]. The high spatial and temporal resolution, high sensitivity and specificity, and operation simplicity of molecular imaging make it a popular choice among researchers [29–32]. Fluorescence imaging, a subtype of molecular imaging, has received much attention in basic research and preclinical applications, particularly for RA diagnosis [33–35].

**Fig. 1:**
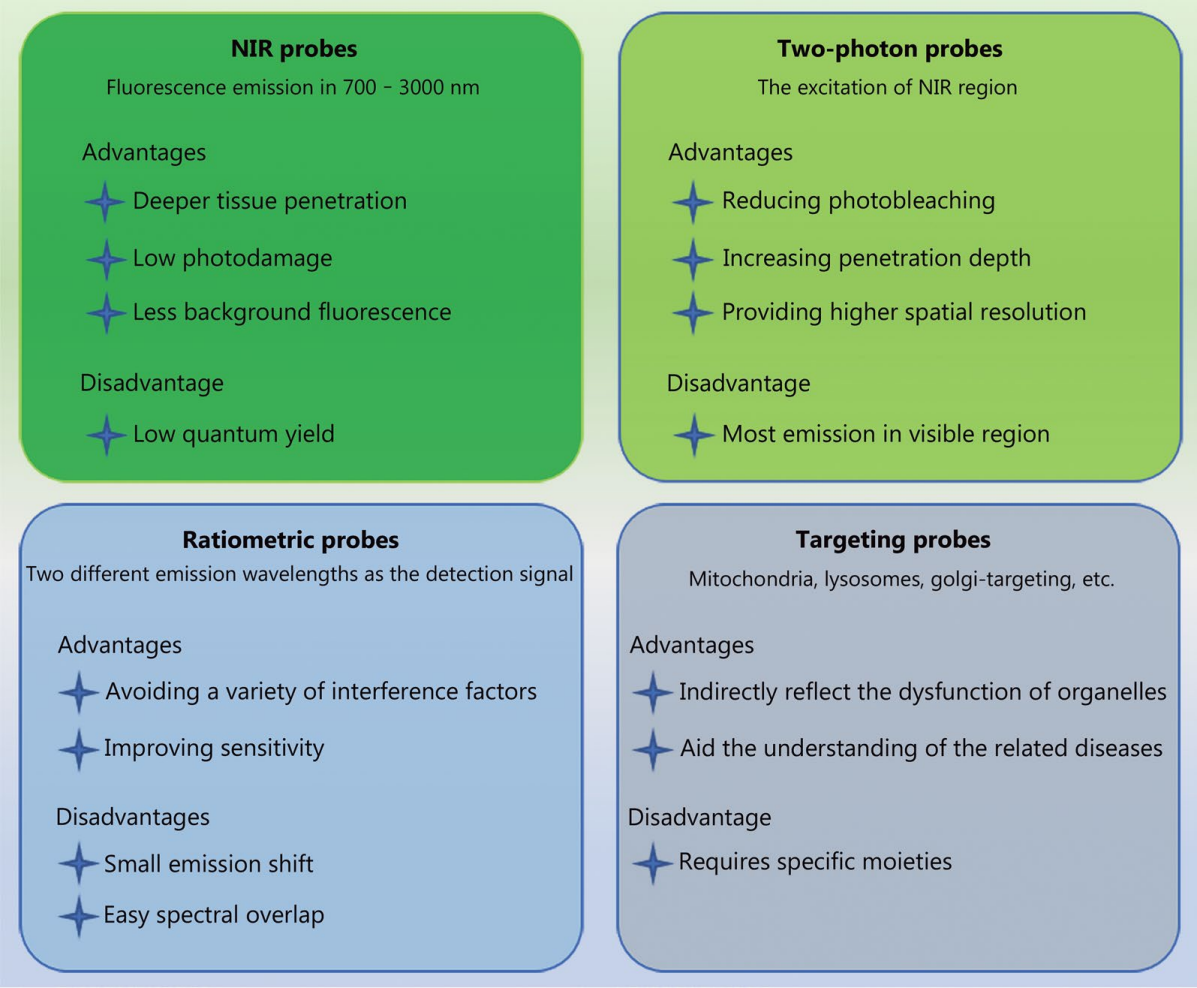
Different features of probes and their advantages and disadvantages. NIR near-infrared

To date, numerous fluorescent probes have been developed to detect and image RA biomarkers such as reactive oxygen species (ROS) and reactive nitrogen species (RNS); however, the related progress in the pathological study and diagnostic process of RA is limited [36–39]. Unlike those based on computed tomography and MRI, RA detection methods replied on optical imaging have not been reviewed so far. Therefore, progress in fluorescence imaging-based RA research should be comprehensively summarized to improve existing RA therapies and advance early RA diagnosis. This work reviews the optical imaging probes [ratiometric type, two-photon type, near-infrared (NIR) type, and targeting type] used in RA research (Fig. 1), discusses their design strategies, action mechanism, and (dis)advantages, and highlights their diversity, utility, potential challenges, and prospects, thus promoting the further exploration of their potential.

## Fluorescence imaging in RA research

Fluorescence imaging is widely used to visualize important physiological processes and diagnose diseases [40–42] and is well suited for RA detection owing to its high sensitivity and specificity, rapid response, and high resolution. Metabolic disorders induced by external stimuli lead to abnormal polarity and oxidative stress in the inflammatory cell microenvironment [43, 44]. This section classifies RA biomarkers, summarizes the current research status and developmental trends of optical RA imaging, and focuses on the design strategy, mechanisms, and application of probes for the diagnosis of immune abnormalities.

### Fluorescent probes for the detection of hypochlorous acid (HClO) as an RA biomarker

HClO is an exceptionally important ROS that is involved in numerous physiological processes, such as immune responses capable of neutralizing invading pathogens and providing resistance against inflammatory diseases [45–47]. It is endogenously produced via the catalytic peroxidation of Cl^−^ by hydrogen peroxide (H_2_O_2_) (mainly through the action of activated neutrophil myeloperoxidase) [48, 49]. Given that high ROS levels, particularly excess HClO, are thought to be associated with the pathogenesis of many inflammatory diseases [50]. In other words, HClO are considered an important marker of RA. To date, numerous types of fluorescent probes have been developed for the bioimaging and environmental detection of HClO. The sensing mechanisms of these probes include the oxidation of substituted phenolic analogs, pyrroles, phenol analogs, thioethers, oximes, or selenides and the cleavage of sulfur or thiolactone, N,N-dimethylthiocarbamate, and C=N or C=C bonds [51–54]. This section describes the fluorescent probes used to detect HClO as a biomarker of RA.

#### Small-molecule fluorescent probes for detecting HClO as an RA biomarker

##### Small molecule fluorescent probes at short wavelengths (< 650 nm)

**Fig. 2:**
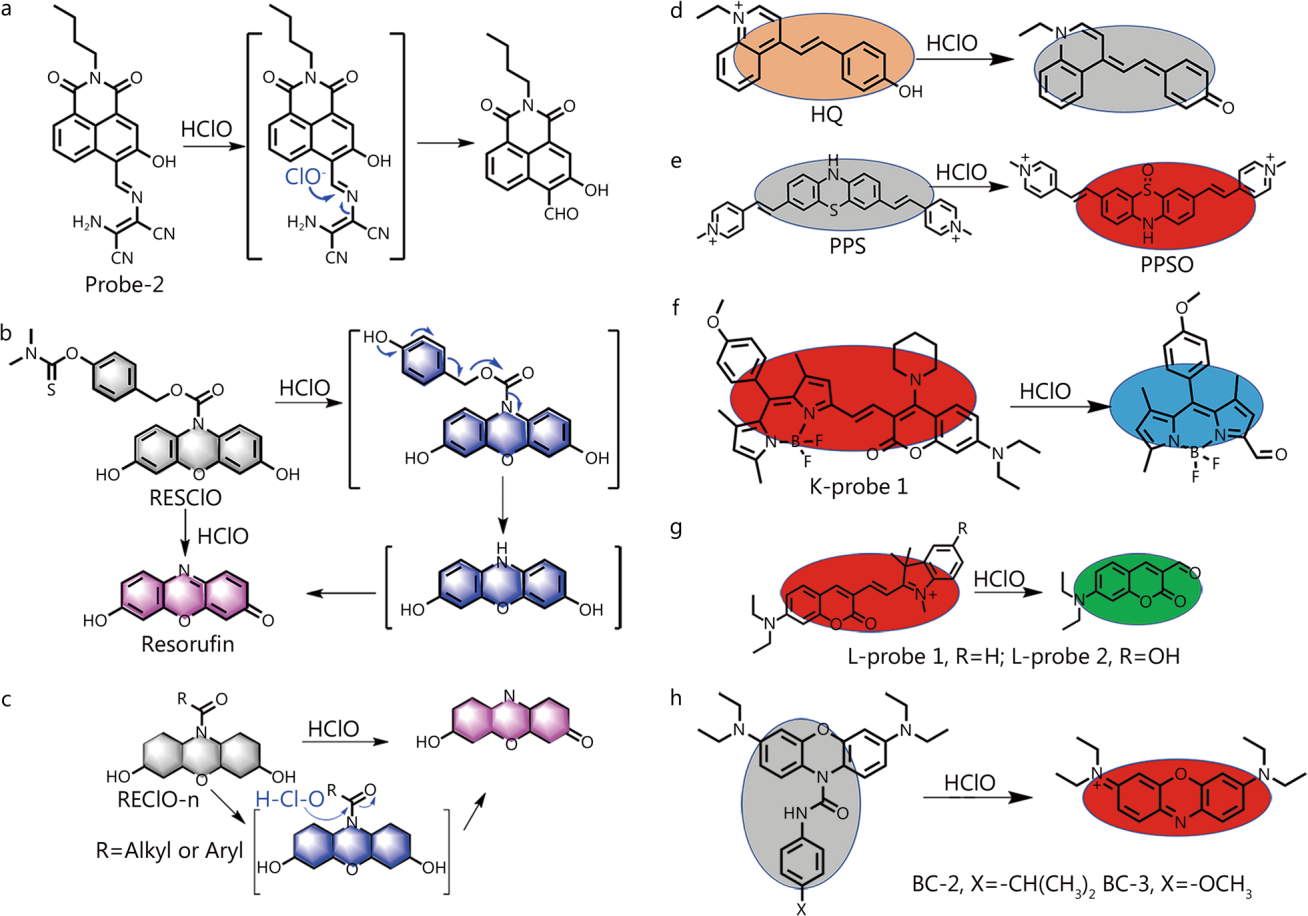
Chemical structure and response mechanism of the probes towards HClO. **a** Schematic illustration of the design strategy and sensing mechanism of probes for monitoring HClO mediated RA. Chemical structure of RESClO (**b**), REClO-n (**c**) and its reaction with HClO. The construction principle and response mechanism of probes HQ (**d**), PPS (**e**), K-probe 1 (**f**), L-probe 1 and 2 (**g**), BC-X (**h**). HClO hypochlorous acid, RA rheumatoid arthritis

 Changes in the cellular levels of endogenous species are often accompanied by disease development [36]. Feng et al. [55] designed and synthesized two fluorescent probes for the visualization and quantitative detection of HClO-induced inflammatory diseases (Fig. 2a), relying on HClO-triggered C=N bond cleavage as a fluorescent signal activator and achieving rapid responses, high specificity and sensitivity, and good biocompatibility. When HClO was present, it specifically triggered C=N bond cleavage, activating the fluorescent signal in 575 nm. Probe-2 enabled the detection of HClO content changes in inflammatory cells, zebrafish with lipopolysaccharide (LPS)-induced inflammation, and mice with arthritis. Specifically, this probe was used to efficiently assess the response of HClO-mediated RA in mice to treatment with methotrexate (MTX), an antiarthritis drug. These results form the basis for future studies on the early diagnosis of RA and the monitoring of HClO-mediated inflammatory diseases.

To further improve detection sensitivity, Wang et al. [56] designed the HClO-specific triggering of a self-immolative fluorescent probe (RESClO) with a trihalocin structure acting as the parent body and an N, N-dimethylthiocarbamate moiety acting as the response group (Fig. 2b). In the presence of HClO, the amide bond was specifically oxidized and broken, thus restoring the red fluorescence of the test halogenated dyes in 590 nm with extensive π-coupling structures. The probe could respond to HClO quickly and demonstrated high sensitivity (16.8 nmol/L). RESClO was used for HClO imaging in cells and zebrafish but was also suitable for the imaging of different HClO levels in animal RA models, thus potentially enabling the early diagnosis and treatment of HClO-mediated diseases. Subsequently, the same group proposed a resorufin-based HClO fluorescent probe (REClO-n) based on HClO-specific oxidative cleavage (Fig. 2c) [57]. Compared to the previously others reported N-protected methylene blue–based HClO probe [46], REClO-n has a different acyl group on the N atom of the dye and therefore features preserved hydroxyl groups and better water solubility. Moreover, the related limit of detection (LOD) was as low as 12 nmol/L, and the linear range (0–20 µmol/L) was larger than that of RESClO. To further improve the LOD, Yang et al. [58] designed a new fluorescent probe that exploited the high oxidizing power of HClO (Fig. 2d) and displayed the lowest LOD (6.5 nmol/L) among the probes reported for HClO monitoring. The phenol group of HQ was oxidized to benzoquinone upon contact with HClO, which in turn led to a burst in the fluorescence signal in 550 nm, thus enabled HClO detection. Considering the excellent spectral properties of this probe, it was employed in the fluorescence imaging of HClO in zebrafish and mouse models. Moreover, it also successfully demonstrated an ability to monitor HClO production in an RA mouse model with and without MTX treatment.

##### Mitochondria-targeted fluorescent probes

 Mitochondria are involved in numerous physiological processes, such as energy production and are essential for maintaining intracellular redox homeostasis [59]. Therefore, it is urgent to develop probes that can be widely applied for the detection of mitochondrial biomarker species. Wu et al. [60] proposed a mitochondria-targeting small-molecule fluorescent probe (PPS) for HClO detection (Fig. 2e). PPS, which focus on the oxidation of sulfur atoms in the phenothiazine core by HClO to sulfoxide moieties and the resulting fluorescence signal enhancement, could sensitively detect endogenous HClO in the study of RA while showing enhanced fluorescence in an acidic environment. Thus, the above work is thought to facilitate research on the role of HClO in the acidic synovial cavity of patients with RA.

##### Ratio-based fluorescent probes

 Ratiometric fluorescent probes facilitate quantitative analysis by allowing the determination of the intensity ratio of signals at two wavelengths, which effectively helps to (i) avoid interference due to uneven probe loading or distribution and (ii) increase the signal-to-noise ratio [61]. Kang et al. [62] prepared a near-infrared (NIR) ratiometric fluorescent probe (K-probe 1) by combining 10-[4-(2,5-dioxo-2,5-dihydro-1 H-pyrrol-1-yl)phenyl]-5,5- (BODIPY) and coumarin aldehyde (Fig. 2f). The conjugated coumarin-BODIPY probe experienced an increase in emission wavelength due to intramolecular charge transfer (ICT). In the presence of HClO, the C=C bond is broken, which leads to the disappearance of red fluorescence and a green fluorescent signal, resulting in a ratiometric detection of HClO. This probe, which exhibited high specificity and a fast response to HClO, was used to image exogenous/endogenous HClO in HeLa cells and a rat model of RA but suffered from suboptimal LOD (0.12 µmol/L). To remedy this deficiency, Lan et al. [63] constructed an OH-substituted coumarin semicarbazone probe (L-probe 2) to ratiometrically monitor subtle HClO content changes in organisms (Fig. 2g). L-probe 2 exhibited excellent specificity and sensitivity (49.1 nmol/L) due to the presence of an electron-donating group (OH) attached to the indole ring. As in the case above, HClO disrupted the conjugated structure of the probe and thus blocked the ICT mechanism, leading to the disappearance of red fluorescence and the emergence of green fluorescence, which formed the basis for ratiometric detection. Moreover, the probe was well capable of penetrating cell membranes and was therefore suitable for the imaging of HClO in mitochondria. L-probe 2 was used to monitor exogenous HClO in active cells and image HClO in keratocystin- and LPS-induced arthritis, providing a good chemical tool for studying the relationship between HClO and arthritic diseases.

##### NIR fluorescent probes

 The short wavelength light emitted by fluorescent probes is easily absorbed by biomolecules, which significantly affects the collection of fluorescent signals in deep tissues [64, 65]. In contrast, emissions in the NIR region (650–3000 nm) offer the advantages of low photodamage and low background autofluorescence in biological samples, and are therefore increasingly favoured by numerous researchers. Based on this concept, Zheng et al. [66] developed two phenoxazine-based NIR probes (BC-2 and BC-3) to monitor and image HClO (Fig. 2h). The fluorescence signal was selectively activated by HClO and not by other ROS. According to the proposed mechanism, HClO attacks the electron-deficient carbon of the amide carbonyl group to cleave the amide moiety and release reduced phenoxazine, which is rapidly oxidized to produce a fluorophore. Probe BC-3 was specifically used to detect endogenous HClO in active cells and animal models of RA.

**Fig. 3:**
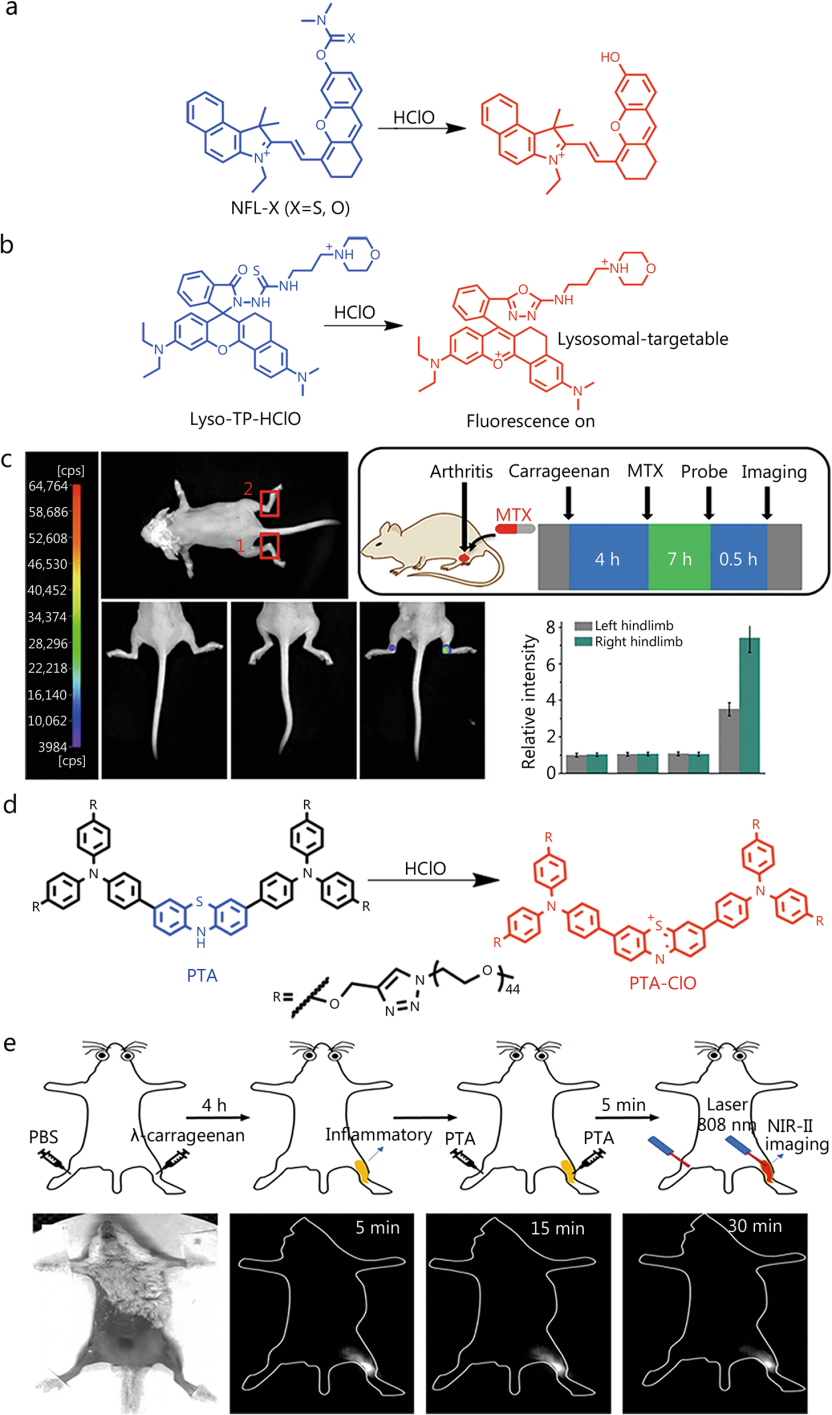
69Structure and application of NIR fluorescent probes for detecting HClO. Structure and response mechanism of the probes, NFL-X (**a**), Lyso-TP-HClO (**b**) and PTA (**d**). **c** Monitoring of response of HClO-induced RA via Lyso-TP-HClO under the treatment with λ-carrageenan or MTX. (1) left hindlimb and (2) right hindlimb. Reproduced with permission from ref. [69]. Copyright (2021) Elsevier B.V. **e** In vivo NIR-II fluorescence imaging of endogenous HClO production in RA mice. NIR-II fluorescence images in 5, 15, and 30 min after the injection of developed probe (PTA). Reproduced with permission from ref. [72]. Copyright (2021) American Chemical Society. HClO hypochlorous acid, NIR near-infrared, MTX methotrexate, RA rheumatoid arthritis, NIR-II second near-infrared region

To increase the emission wavelength, reduce autofluorescence, and enhance tissue penetration, Qian et al. [67] designed two fluorescent probes (NFL-S and NFL-O) with hemiflowered cyanocyanine backbones and thiocarbamate response groups (Fig. 3a). NFL-S showed high selectivity for HClO, which induced thiocarbamate release and ICT mechanism restoration to afford a clear fluorescent signal. This probe was useful for detecting endogenous HClO released from RAW 264.7 cells and could be employed to monitor HClO in LPS-stimulated RA mouse models.

Lysosomes are important cellular organelles that may induce apoptosis when invaded by external substances [68], which highlights the importance of detecting endogenous species in lysosomes. A two-photon NIR fluorescent probe (Lyso-TP-HClO) capable of targeting lysosomes and aggregating therein was reported by Mao et al. [69] (Fig. 3b). This probe, which featured morpholine groups as lysosomal targeting units, exhibited high selectivity and a wide HClO response range. Owing to the two-photon-excited NIR emission, Lyso-TP-HClO exhibited a small fluorescence background and high penetration depth and was deemed suitable for monitoring endogenous HClO in the lysosomes of bacterially infected cells. In addition, this probe was used to detect HClO in a mouse model of RA and evaluate the therapeutic effect of MTX (Fig. 3c). Thus, Lyso-TP-HClO was proposed to be a useful chemical tool for the early diagnosis of RA and treatment response monitoring.

Compared to NIR-I fluorescent probes, NIR-II ones are considered promising for the early diagnosis of some diseases because of their deeper tissue penetration ability, high resolution, small background fluorescence, and reduced scattering [70, 71]. Thus, effective fluorescent probes for the early diagnosis of RA based on these considerable advantages are highly sought after. Wu et al. [72] proposed an NIR-II probe based on phenothiazine and triphenylamine to detect HClO, providing a high-quality tool for early RA diagnosis (Fig. 3d). The probe was designed based on the ICT mechanism. HClO oxidized the electron-rich phenothiazine moiety to the strongly electron-accepting phenothiazin-5-ium ion to produce a D-A-D structure with a strong fluorescence signal at 936/1237 nm. The probe selectively responded to HClO within 30 s and exhibited good water solubility and photostability. Most importantly, endogenous HClO was rapidly visualized using the fluorescence signal from this NIR-II probe in an inflammatory RA mouse model with a 4.3-fold signal enhancement. Thus, this study provides a valuable tool for early diagnosis of RA (Fig. 3e).

#### **Nanofluorescent probes for the detection of HClO as an RA biomarker**

**Fig. 4:**
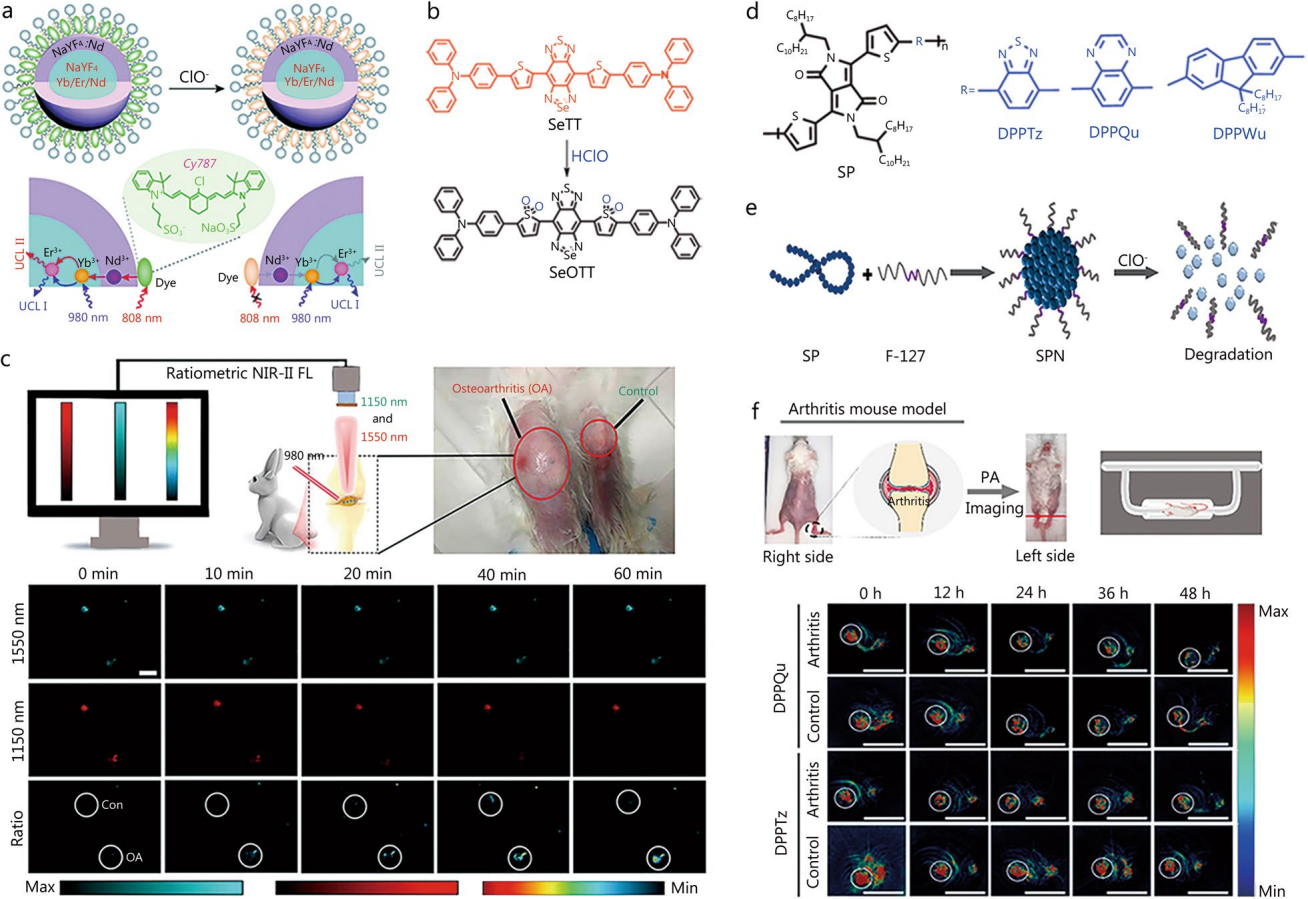
Structure and application of nanofluorescent probes for detecting HClO. **a** Schematic of dye-sensitized UCNP nanocomposites for the UCNP ratiometric probing of HClO and UV-vis absorption. Reproduced with permission from ref. [75]. Copyright (2019) Royal Society of Chemistry. **b** Structure and response mechanism of the probe SeTT toward HClO. **c** NIR-II ratiometric fluorescence imaging of osteoarthritis in rabbits using the nanoprobe. Reproduced with permission from ref. [77]. Copyright (2020) American Chemical Society. **d** Structure of the probes of DPPTz, DPPQu, and DPPWu. **e** Formation process of nanoprobes. **f** Imaging of HClO in λ-carrageenan-induced arthritic mice. **d, e** and **f** were reproduced with permission from ref. [81]. Copyright (2022) American Chemical Society. HClO hypochlorous acid, NIR near-infrared, UCNP upconversion nanoparticles, UV-vis ultraviolet-visible, PA photoacoustic, ClO^−^ hypochlorite ion, NIR-II second near-infrared region, FL fluorescence imaging, UCL luminescence emission signal

Lanthanide-doped upconversion nanoparticles (UCNPs) exhibit the advantages of large Stokes shifts, high photostability, zero autofluorescence background, and high penetration depth [73, 74]. Consequently, the corresponding nanoprobes hold great promise for cellular and in vivo imaging. Inspired by the advantages of nanoprobes, Zou et al. [75] developed a dual-excitation (980 and 808 nm) single-emission ratiometric fluorescent probe (UCNPs-Cy787@PC) using a dye-sensitized upconversion strategy (Fig. 4a). In this design, the Cy787 dye acts as an energy donor and a specific recognition element, and HClO suppresses the fluorescence signal under 808 nm excitation without altering the fluorescence signal under 980 nm excitation, which enables ratiometric detection. UCNPs-Cy787@PC exhibited high selectivity for HClO while featuring the advantages of high stability and good biocompatibility. This probe has been widely used to detect HClO in arthritic mice and perform semiquantitative analyses, thus holding great promise for the study of HClO-related inflammatory diseases. Although UCNPs-Cy787@PC exhibited excellent HClO detection performance, its further applications are hindered by its overly short emission wavelength. To address this drawback, Ma et al. [76] developed a long-lived Eu^3+^-based luminescent probe for the rapid monitoring of HClO in mitochondria, which are responsible for numerous physiological processes such as the maintenance of intracellular redox homeostasis. Surprisingly, the developed probe exhibited a rapid response (5 s) and high specificity for HClO and mitochondrial aggregation, thus holding promise for the luminescence imaging of mitochondrial HClO as a biomarker of RA. In addition, it can be employed to monitor HClO levels in animal models of liver injury and RA and was successfully embedded into a smart sensor film capable of monitoring wound infections and acting as a valuable tool to facilitate the diagnosis of such diseases.

To improve tissue penetration, Ge et al. [77] developed an NIR-II fluorescent probe (SeTT) with a selective response to HClO (Fig. 4b) relying on a rapid fluorescence intensity loss at 1150 nm. Compared with switch-type fluorescent probes, ratiometric ones are more popular because of their high sensitivity and lack of dependence on environmental factors. The above ratiometric probe with dual-wavelength emission upon single-wavelength excitation delivered a more accurate signal than the probe with single-wavelength emission upon dual-wavelength excitation because of the reduced attenuation difference in the former/latter case. The NIR-II ratiometric probe, produced by encapsulating SeTT in UCNPs, was used to detect fluctuations in HClO content during tumorigenesis and monitor HClO content in the peritoneal cavity of inflamed mice and in an arthritic rabbit model (Fig. 4c). Commercially available probes are more readily available than those that require synthesis. Cao et al. [78] synthesized a ratiometric NIR-II fluorescent probe for HClO detection using a commercially available organic dye (Cy925) and Er^3+^-doped UCNPs. UCNP fluorescence emission was used as a reference, whereas Cy925 was used as a response molecule for HClO, which enabled the ratiometric detection of the latter, as exemplified by the dynamic detection of HClO content changes in arthritic mouse limbs.

Semiconductor polymers have relatively high extinction coefficients and can be served as contrast agents for imaging [79, 80]. Unfortunately, some semiconductor polymers (e.g., nonbiodegradable semiconductor materials) can remain in the body and thus pose health risks. Therefore, biodegradable semiconductor materials for the detection and imaging of in vivo biomarkers are highly sought after. Ma et al. [81] developed three biodegradable semiconductor nanoprobes (DPPTz, DPPQu, and DPPWu) based on diketopyrrolopyrrole-derived polymers for HClO detection (Fig. 4d). DPPQu and DPPTz nanoprobes are typical photoacoustic imaging contrast agents with many advantages. They were applied to study LPS-stimulated changes in endogenous HClO levels and examine the fluctuating levels of HClO by photoacoustic imaging in an animal model of HClO-related RA in situ (Fig. 4e, f). Table 1 summarizes the fluorescent probes used for HClO detection.

**Table 1:** Fluorescence probe for imaging HClO as an RA biomarker

Sensor	λ_em_ (nm)	Signal type	Targeted site	Response time	LOD	Biological applications	References
Probe-2	575	Turn-on	–	4 s	17.3 nmol/L	Exogenous HClO imaging in cells, endotoxin-induced inflammation in adult zebrafish and RA in mice with an antiarthritic drug (MTX)	[55]
RESClO	590	Turn-on	–	10 s	16.8 nmol/L	Exogenous HClO imaging in cells, zebrafish and mice arthritis models	[56]
REClO-n	590	Turn-on	–	50 s	12 nmol/L	HClO-mediated arthritis and solid tumour mouse models	[57]
HQ	550	Turn-off	–	25 s	6.5 nmol/L	Exogenous and endogenous HOCl imaging in cell, zebrafish and RA mouse models with and without MTX treatment	[58]
PPS	580	Turn-on	Mitochondria	100 s	24 nmol/L	Endogenous HClO imaging in mitochondria and a λ-carrageenan induced RA mouse model; mitochondria colocalization experiments	[60]
K-probe 1	510/660	Ratiometric	–	0.5 s	0.12 µmol/L	HClO imaging in living cells and an arthritis nude mouse model	[62]
L-probe 2	500/650	Ratiometric	–	2 min	49.1 nmol/L	Exogenous and endogenous HClO imaging in living cells and λ-carrageenan/LPS- induced arthritis models	[63]
BC-3	669	Turn-on	–	50 s	11.1 nmol/L	Exogenous HClO imaging in living cells in a rheumatoid arthritis mouse model	[66]
NFL-S	732	Turn-on	–	30 s	35 nmol/L	Endogenous HClO imaging in RAW 264.7 macrophage cells and in an LPS-stimulated arthritis mouse model	[67]
Lyso-TP-HClO	655	Turn-on	Lysosome	2 min	30 nmol/L	HClO imaging in RAW 264.7 cells and a λ-carrageenan-induced arthritis model with and without MTX treatment; lysosome-targetable colocalization experiments	[69]
PTA	936/1237	Turn-on	Lysosome	30 s	55 nmol/L	Exogenous and endogenous HClO imaging in living cells and an inflammatory RA model; lysosome colocalization	[72]
UCNPs-Cy787@PC	540	Turn-on	–	–	3.6 nmol/L	HClO imaging in an arthritis mouse model	[75]
Eu(L)_3_(DPBT)	607	Turn-off	Mitochondria	5 s	14.7 nmol/L	Monitoring of HClO levels in mimicked inflammatory cells, endotoxin-induced liver injury, rheumatoid arthritis in live mice and mouse wounds; mitochondria colocalization experiments	[76]
DCNP@SeTT@PEG	1150/1550	Ratiometric	–	2 min	0.4 µmol/L	Visualizing and imaging HClO in arthritis, peritoneal cavity inflammation, tumours in mice, and osteoarthritis in rabbits	[77]
Er-CSSNPs@Cy925	925/1550	Ratiometric	–	6 min	–	Imaging HClO in the formation of inflammation in mice hind limbs	[78]
DPPTz NPs/DPPQu NPs/DPPWu NPs	550	Turn-on	–	12 h	–	Visualizing and imaging HClO in LPS-stimulated RAW 264.7 cells and a λ-carrageenan-induced arthritis mouse model	[81]

*HClO* hypochlorous acid, *RA* rheumatoid arthritis, *LPS* lipopolysaccharide, *LOD* limit of detection, *MTX* methotrexate

### Fluorescent probes for the detection of peroxynitrite (ONOO^−^) as an RA biomarker

ONOO^−^, an RNS with high oxidation and nitration activities, is associated with several dominant pathological activities [82, 83]. This species, which has a relatively short half-life and is mainly produced from NO and superoxide anion radicals under free-diffusion conditions [25], can react with many substances such as DNA to cause apoptosis or even cell death [84, 85] and is related to pathological processes such as inflammation, tumorigenesis, and autoimmune diseases [86]. For example, ONOO^−^ is overproduced during RA development. To understand the key functions of ONOO^−^ in various diseases, one should develop rapid and effective probes for detecting changes in ONOO^−^ levels in living organisms. Here, we summarize the fluorescent probes recently developed to detect ONOO^−^ as a biomarker of RA.

**Fig. 5:**
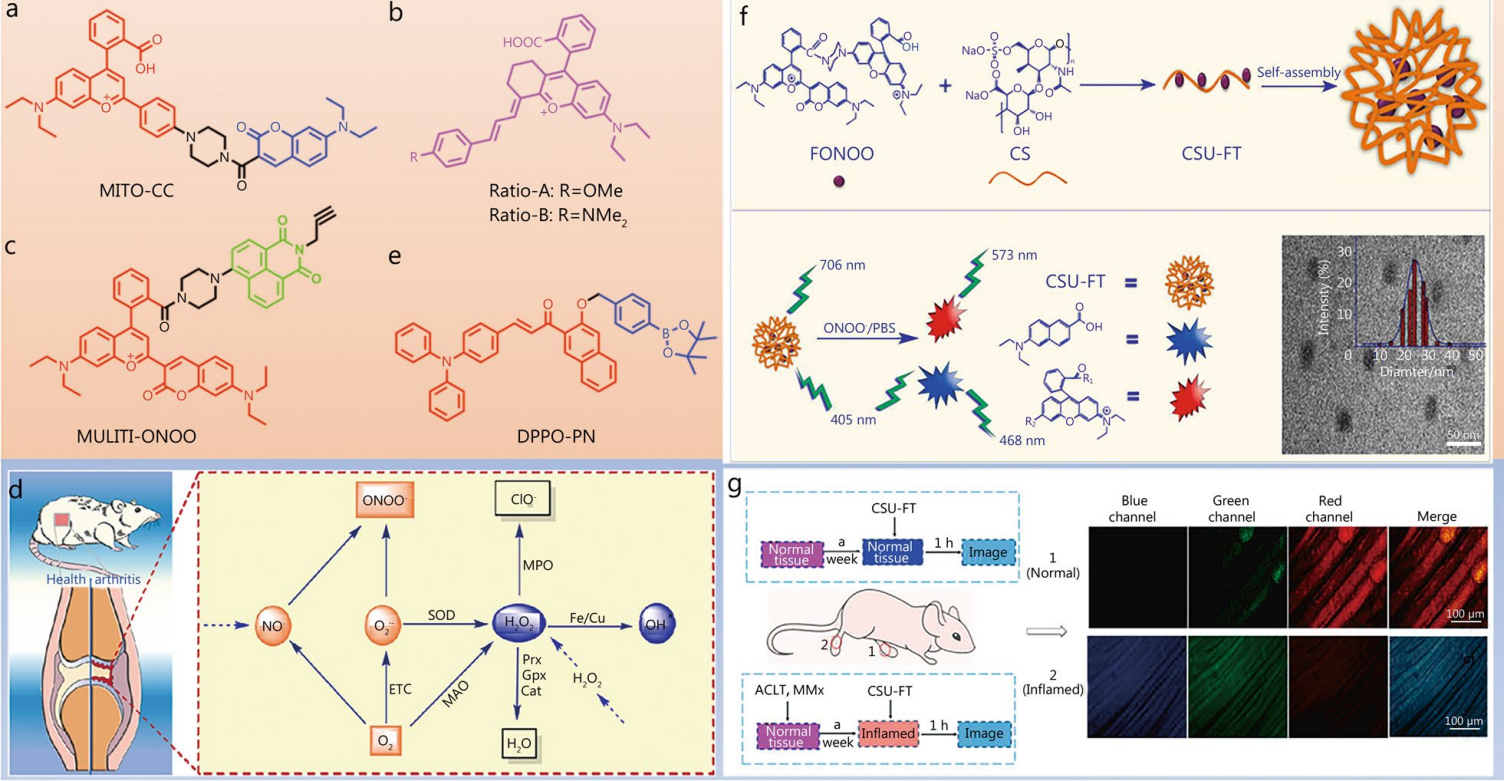
Structure and application of NIR fluorescent probes for detecting ONOO^−^. Structures of the probes MITO-CC (**a**), Ratio-R (**b**), MULTI-ONOO (**c**) and DPPO-PN (**e**). **d** Mechanism of ONOO^−^ production in arthritis diseases developments. Reproduced with permission from ref. [89]. Copyright (2022) Elsevier B.V. **f** Structure and ONOO^−^-sensing mechanism of the nanoprobe CSU-FT. **g** Fluorescence microscopy imaging of inflamed tissue with CSU-FT. **f** and **g** were reproduced with permission from ref. [94]. Copyright (2022) Elsevier B.V. ONOO^−^ peroxynitrite, NIR near-infrared, NO nitric oxide, O_2_^·−^ superoxide anion, O_2_ oxygen, SOD superoxide dismutase, ETC electronic transfer of charge, MAO monoamineoxidase, H_2_O_2_ hydrogen peroxide, H_2_O water, MPO myeloperoxidase, ClO^−^ hypochlorite ion, Prx peroxiredoxin, Gpx glutathione peroxidase, Cat catalase, ·OH hydroxyl radicals, Fe ferrum (iron), Cu cuprum (copper), PBS phosphate buffer saline, ACLT anterior cruciate ligament transection, MMx medial meniscus resection

To understand the role of ONOO^−^ in arthritis development, Cheng et al. [87] synthesized a ratiometric two-photon fluorescent probe (MITO-CC) for the rapid monitoring of ONOO^−^ in organisms based on fluorescence energy resonance transfer (FRET) (Fig. 5a). Due to its nucleophilicity and oxidative power, ONOO^−^ may undergo nucleophilic addition, oxidation, elimination, and hydrolysis reactions to eventually generate olefinic acid products, disrupt the FRET mechanism, and cause green fluorescence. Compared to single-photon fluorescent probes, two-photon ones have a longer excitation wavelength and therefore cause less damage to biological tissues. The above probe not only exhibited a fast response (20 s) and low LOD, but also selectively detected ONOO^−^ in the presence of other ROS or RNS. ONOO^−^ level fluctuations were detected using MITO-CC and single- and two-photon confocal microscopy. Most importantly, MITO-CC could be used to detect spike fluctuations in ONOO^−^ levels in an LPS-induced joint inflammation mouse model, providing a new approach for studying the relationship between ONOO^−^ and related diseases.

To achieve a faster response to ONOO^−^, Lu et al. [88] prepared two ratiometric NIR fluorescent probes (Ratio-A and Ratio-B) for the detection of this marker in biological systems (Fig. 5b). The butadienyl bridge, which can be oxidized by ONOO^−^ to form ketones and thus induce green fluorescence accompanied by large Stokes shifts and effectively avoid spectral overlap, was chosen as the response group for ONOO^−^. Ratio-A could not only detect the change in ONOO^−^ content in RAW 264.7 cells, but also enabled ONOO^−^ monitoring in an animal RA model. In addition, changes in ONOO^−^ levels during MTX treatment were evaluated to explore the role of ONOO^−^ in inflammatory diseases and provide a deeper understanding of disease development.

Compared with monochrome imaging, multichannel imaging can effectively avoid environmental factor-related errors and effectively improve detection precision. Xu et al. [89] reported a multichanneled ratiometric-based fluorescent probe (MULTI-ONOO) for the visualization of ONOO^−^ levels in cells and in a mouse arthritis model (Fig. 5c), which had the advantage of providing multichannel information and being more suitable for bioimaging in multicomponent complex environments compared to monochrome imaging. MULTI-ONOO consisted of a naphthalenedicarboximide fluorophore and a NIR xanthene fluorophore, and was used to detect ONOO^−^ in multiple colours by a FRET mechanism. It exhibited a short response time (approximately 20 s) and LOD of 11.6 nmol/L for ONOO^−^ and could be applied in complex environments for multicomponent analysis. The probe was successfully used to detect ONOO^−^ in live cells, arthritic tissues and rat models (Fig. 5d).

Aggregation-induced emission (AIE) probes can effectively overcome the disadvantages of aggregation-induced burst probes, especially in terms of inaccuracies caused by different local probe concentrations and detection accuracy owing to the inhomogeneous distribution of probes in the intracellular environment [90, 91]. Zhang’s group [92] designed activatable AIE fluorescent probes (DPPO-PN) for the detection of ONOO^−^ in organisms (Fig. 5e). An AIE active part and an excited-state intramolecular proton transfer (EISPT) active part were integrated into the probe, and benzoborate was used as a sensitive response site for ONOO^−^. In the presence of ONOO^−^, EISPT was activated and resulted in bright fluorescence. DPPO-PN could rapidly respond to ONOO^−^ (30 s), experiencing an approximately 161-fold fluorescence signal enhancement, and was used to quantify traces of ONOO^−^ in RAW 264.7 cells and animal models of LPS/CFA-induced RA.

Compared to fluorescent probes located in the visible region as well as NIR-I, NIR-II fluorescent probes exhibit deeper tissue penetration, higher resolution, and less pronounced autofluorescence and are therefore considered more suitable for imaging biological tissues [71]. To increase the application potential of NIR-II probes for the detection of ONOO^−^ in living organisms and shed light on the pathogenesis of RA, Wang’s group [93] designed a NIR-II nanoprobe based on lanthanide-doped downshifting nanoparticles. The NaErF_4_@NaYF_4_@NaYF_4_:10%Nd@NaYF_4_ probe emitted intense fluorescent signals at 1060 and 1525 nm upon excitation at 808 nm. After coupling with dye A1094, the fluorescence signal at 1060 nm was quenched. ONOO^−^ activated emission at 1060 nm without affecting the signal at 1525 nm, which enabled the ratiometric detection and the efficient and rapid imaging of this marker. Moreover, the probe could effectively image the NIR-II window in a practical application process, which can promote the further application of fluorescence detection technology. Although the above probe is suitable for deep tissue imaging in living organisms, its detection limit is relatively low. To improve detection sensitivity and avoid the influence of environmental factors, Zhou’s group [94] designed a multicolour fluorescent nanoprobe to detect ONOO^−^ for the early diagnosis of arthritis therapy effects and the qualitative and quantitative detection of ONOO^−^ during the development of inflammatory diseases. The developed nanoprobe (CSU-FT) featured a FRET-based action mechanism and was prepared by coupling rhodamine B to xanthan followed by grafting onto chondroitin sodium (Fig. 5f). CSU-FT, which exhibited a short response time (< 20 s) and excellent LOD (11.7 nmol/L), was used to detect ONOO^−^ and probe the diagnostic and therapeutic effects on RA in rats, providing results indicative of a link between ONOO^−^ and arthritis. Unfortunately, this probe did not allow a deeper study of arthritis therapy effects (Fig. 5g), which presents a new challenge for researchers. Table 2 summarizes the fluorescent probes used to detect ONOO^−^.

**Table 2:** Fluorescence probe for imaging ONOO^−^ as an RA biomarker

Sensor	λ_em_ (nm)	Signal type	Targeted site	Response time	LOD	Biological applications	References
MITO-CC	473/651	Ratiometric	Mitochondria	20 s	11.3 nmol/L	Exogenous ONOO^−^ imaging in cells and an LPS-stimulated inflamed mouse model; mitochondria colocalization experiments	[87]
Ratio-A	564/700	Ratiometric	–	10 s	28.06 nmol/L	Exogenous ONOO^−^ imaging in RAW 264.7 cells and arthritis mouse models	[88]
MULTI-ONOO	468/526/706	Ratiometric	–	20 s	11.6 nmol/L	Exogenous ONOO^−^ imaging in RAW 264.7 cells, arthritis tissues and arthritis mouse models	[89]
DPPO-PN	632	Turn-on	–	30 s	10 nmol/L	Exogenous ONOO^−^ imaging in RAW 264.7 cells, HeLa cells and arthritis mouse models	[92]
DSNP@A	1060/1525	Ratiometric	–	–	0.8 µmol/L	Endogenous ONOO^−^ imaging in an autoimmune induced RA mouse model	[93]
CSU-FT	468/576/706	Ratiometric	–	20 s	11.7 nmol/L	Exogenous ONOO^−^ imaging in RAW 264. 7 cells and arthritis mouse models	[94]

*RA* rheumatoid arthritis, *LPS* lipopolysaccharide, *LOD* limit of detection, *ONOO*^*−*^ peroxynitrite

### Fluorescent probes for the detection of other ROS as RA biomarkers

In addition to HClO and ONOO^−^, cells contain other ROS molecules, such as H_2_O_2_, hydroxyl radical (·OH), and nitroxyl (HNO) [95]. ROS can greatly impact cell signalling and thus influence physiological and pathological processes such as cellular metabolism and differentiation [96]. An abnormal increase in cellular ROS levels can cause oxidative stress, destroy cell structure, and even lead to cell dysfunction and apoptosis in severe cases [97, 98]. Therefore, ROS are the starting point for the treatment of various ROS-induced diseases. This section reviews the ROS (except HClO and ONOO^−^) used in RA treatment.

#### Detection of HNO as an RA biomarker

**Fig. 6:**
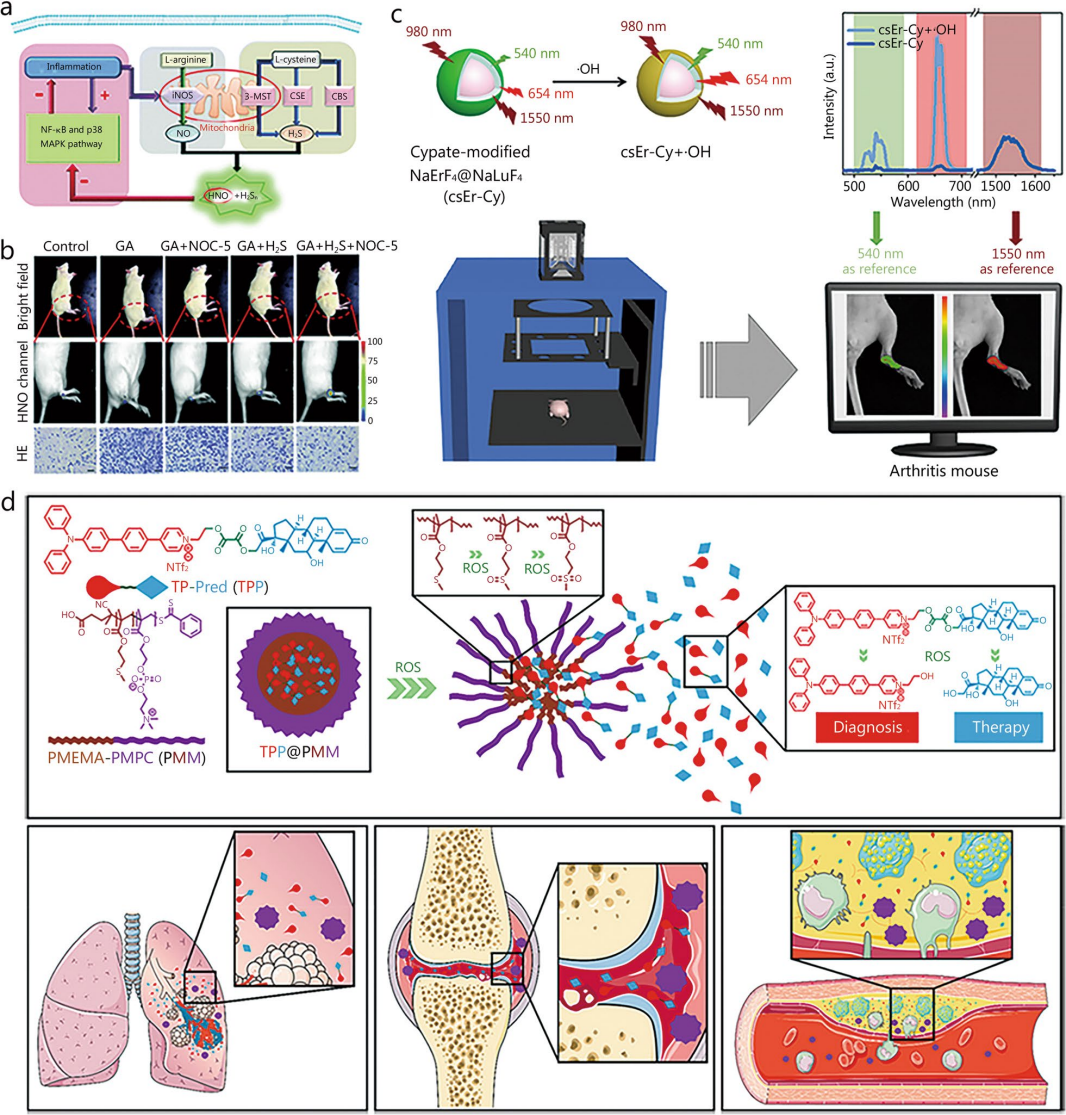
Structure of ROS-related probes and their applications. **a** Illustration of the generation of endogenous HNO and H_2_S_n_ in living cells. **b** Representative in vivo NIR fluorescence images visualizing HNO in a gouty arthritis rat model. **a** and **b** were reproduced with permission from ref. [99]. Copyright (2019) Royal Society of Chemistry. **c** Schematic illustration of the interference-free ·OH detection mechanism of the csEr-Cy nanoprobe and its application in vivo. Reproduced with permission from ref. [100]. Copyright (2022) American Chemical Society. **d** Illustration of the development of a nanoplatform with two-photon imaging and serial ROS sensitivity. Reproduced with permission from ref. [101]. Copyright (2022) American Chemical Society. ROS reactive oxygen species, NO nitroxyl, H_2_S_n_ polysulfide, ·OH hydroxyl radical, 3-MST 3-mercaptopyruvate sulfurtransferase, CSE cystathionine γ-lyase, CBS cystathionine-β-synthase, HE hematoxylin-eosin, HNO nitroxyl, GA gouty arthritis, a.u. arbitrary unit, NTf_2_ bis(trifluoromethanesulfonyl)imide, H_2_S hydrogen sulfide, NOC-5 3-[2-hydroxy-1-(1-methylethyl)-2-nitrosohydrazinyl]-1-propanamine, iNOS inducible nitric oxide synthase

HNO exacerbates ischemia-related injury, induces neurotoxicity, and is associated with the development of RA. Although several fluorescent probes have been developed to detect HNO, few of them are suitable for HNO detection during RA progression. To investigate the relationship between HNO and RA disease, Chen’s group [99] synthesized a mitochondria-targeting NIR fluorescent probe (Mito-JN) for the assessment of HNO levels in gouty arthritis development. Aza-BODIPY was introduced as the sensor fluorophore, cationic triphenylphosphine was selected as the mitochondria-targeting localization group, and the triarylphosphine group acted as the HNO response unit. The reaction of HNO with triarylphosphine produces phosphine oxide and aza-acyl linkages, which immediately undergo intramolecular ester aminolysis to release the alcohol/amide. Mito-JN exhibited high selectivity for HNO against other biological species as well as low cytotoxicity and was used to detect the endogenous production of HNO to reveal that this species originates from polysulfide (H_2_S_n_) and NO in living cells (Fig. 6a). The anti-inflammatory effect of HNO was detected in an LPS-induced RA cellular model, had a significant impact on the pathological process of the disease, and was confirmed using pathological slices, indicating the high application value of the developed probe in inflammation treatment (Fig. 6b).

#### Detection of ·OH as an RA biomarker

·OH, which is the most harmful ROS because of its high oxidative reactivity, plays an important role in the development of inflammatory conditions such as arthritis and may lead to physiological disorders in biological systems. To further investigate the relationship between ·OH and RA, a novel ring-modified core-shell nanoprobe (csEr-Cy) for the sensitive detection of ·OH was designed and successfully prepared by Zhou’s group [100] (Fig. 6c). In the presence of ·OH, FRET mechanism suppression resulted in the recovery of the fluorescence signal at 654 nm, whereas the NIR-II fluorescence signal at 1550 nm remained unchanged, which enabled the ratiometric interference-free detection of ·OH. The underlying mechanism was probed using density functional theory calculations. csEr-Cy nanoprobes were found to have higher penetration abilities than visible-light reference probes and have been successfully applied to RA diagnosis in mice, providing new insights for improving molecular detection sensitivity.

#### Simultaneous detection of multiple ROS as RA biomarkers

Despite the development of single ROS-targeting fluorescent probes, their selectivity remains suboptimal. It is also critical to develop fluorescent probes that are responsive to multiple ROS for the diagnosis of RA. With this in mind, Ma et al. [101] reported a two-photon AIE nanoprobe capable of a continuous response to ROS. Prednisolone was bridged to the developed two-photon fluorophore to form a diagnostic therapeutic compound, TP-Pred (TPP) (Fig. 6d), which self-assembled with an amphiphilic deblocking copolymer to form polymeric micelles (TPP@PMM). The introduction of poly (2-methacryloyloxyethyl phosphorylcholine) resulted in excellent protein adsorption, satisfactory biocompatibility, and stability during transport in blood because of the amphoteric nature of the phosphorylcholine moiety. Under certain conditions, the micellar structure was interrupted by ROS-triggered poly (2-methylthio ethanol methacrylate), which released TPP. The free TPP bound to overexpressed ROS to result in secondary ROS sensitization. Thus, accurate prednisolone delivery was achieved to substantially improve anti-inflammatory levels, and the related mechanism can be judged accordingly. In view of its two-photon fluorophore and sustained ROS reactivity, the probe featured good anti-inflammatory activity and has been used for the effective diagnosis and treatment of RA. Therefore, TPP@PMM was concluded to be a promising two-photon diagnostic and sequential ROS-triggered therapeutic candidate for the treatment of acute inflammation.

### Fluorescent probes for the detection of pH in RA

**Fig. 7:**
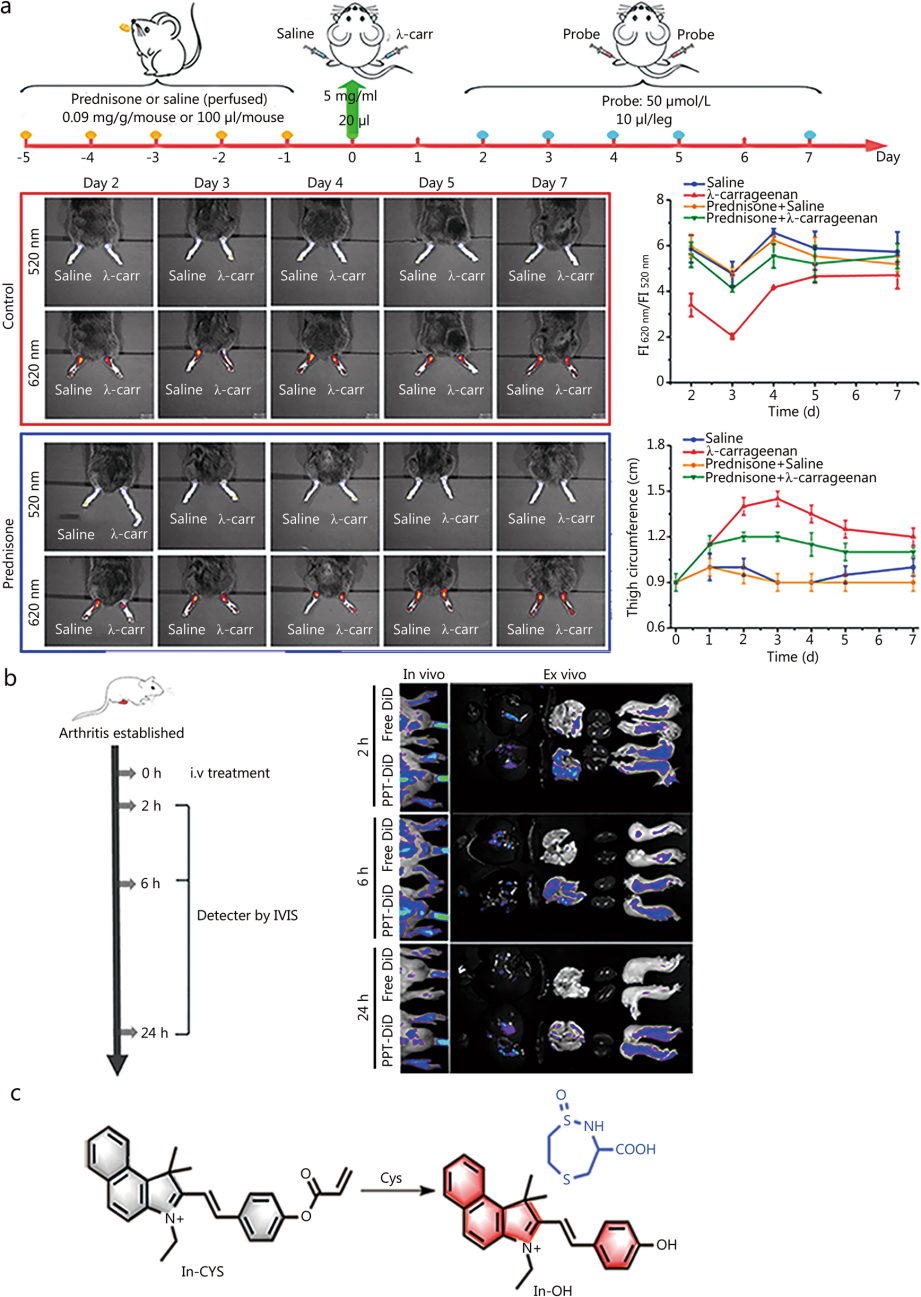
Structure of pH or Cys-related probes and their applications. **a** Fluorescence imaging of pH-ER in a λ-carrageenan-induced RA model. Reproduced with permission from ref. [104]. Copyright (2021) Elsevier B.V. **b** Probe’s (PEG-PBA-TGMS, PPT) application in vivo. Reproduced with permission from ref. [105]. Copyright (2021) American Chemical Society. **c** Mechanism of In-CYS reacts with Cys. Cys cysteine, RA rheumatoid arthritis, λ-carr λ-carrageenan, i.v intravenous injection, IVIS in vivo imaging system, DiD a near-infrared fluorescent dye

Given that pH can significantly influence numerous biological processes such as cell proliferation, translocation, and apoptosis, the regulation of pH and internal homeostasis is essential for cell viability. Under normal physiological conditions, cytoplasmic pH is generally 7.2–7.4; however, there is some variation in the pH of different organelles such as lysosomes (4.5–5.5) and mitochondria (8.0–8.2) [102]. Abnormalities in intracellular pH may lead to pH disturbances throughout the cell, resulting in increased levels of free radicals, inappropriate apoptosis, and necrosis. In addition, abnormal pH can contribute to the development of cancer as well as neurodegenerative, heart, and liver diseases. For example, the microenvironment of tumours is more acidic than that of healthy tissues [103]. Therefore, accurate pH monitoring is important for studying cellular functions and pathological processes. Tang’s team [104] developed a ratiometric fluorescent probe (pH-ER) for probing pH in the endoplasmic reticulum. A benzindole semicarbazide derivative was introduced as a luminescent fluorophore, a hydroxyl group was served as the pH-responsive group, and a methanesulfonamide group was selected as the ER-targeting group. The probe showed remarkable results over a relatively wide pH range (3.98–11.03) and was employed to detect the pH of the reticulum under tunicamycin-induced oxidative stress, study pH fluctuations during dexamethasone-induced apoptosis, and evaluate the efficacy of prednisone acetate in a mouse model of λ-carrageenan-induced RA (Fig. 7a). After the addition of prednisone acetate, RA symptoms were significantly alleviated, and the ratiometric signal was significantly lower than that in normal mice. The development of the above probe provides guidance for assessing the pharmacological effects of various drugs for RA treatment.

Single-effect fluorescent probes are easily affected by environmental factors, whereas multi-effect fluorescent probes exhibit substantially improved detection sensitivity and accuracy. He et al. [105] coupled polyethylene glycol-phenylboronic acid-triglycerol monostearate with self-assembled dual-stimulus-responsive polymer micelles to obtain a dual-responsive micellar nanoparticle probe effectively detecting acidic pH and overexpressed metalloproteinases (PEG-PBA-TGMS and PPT) (Fig. 7b). In the constructed RA model, PPT micelles accumulated in the arthritic joint, and dexamethasone was released at this site to achieve therapeutic effects. The double-stimulatory-response micelles were concluded to be important for the treatment of inflammatory diseases and could promote the improvement of curative effects, thus having high application value for RA treatment. To further investigate the interaction between pH and RA, Liu et al. [106] developed pH-responsive nanoparticles and examined their performance and applicability in the diagnosis of immune disorders. An anti-inflammatory agent, namely tretinoin lactone, was encapsulated in POSS-PCL-b-PDMAEMA to effectively relieve inflammation. Cytotoxicity and apoptosis were significantly reduced in treated RAW 264.7 cells. Another important advantage is that the drug concentration in the target tissue can be detected accurately. Upon the injection of indocyanine green-labeled nanoprobes into a mouse model of arthritis, the above compound exerted a good protective effect on cartilage and significantly reduced the inflammatory response.

### RA was detected by fluorescent probes containing liposome

Lipid droplets as an important organelle and targeting them to detect some endogenous species is of key importance. Wu et al. [17] prepared fluorescent probes targeting lipid droplets modified with iRGD peptides (iLPs) and explored their suitability for fluorescence molecular imaging-based RA detection and evaluation using quantitative analysis. Fifteen minutes after injection, the fluorescence signal in the iLP group was 3.03-fold higher than that in the LP-modified group (*P* < 0.01), while the fluorescence signal of the iLP group was barely visible in the absence of inflammation. iLP-based NIR fluorescence imaging showed high sensitivity and accuracy for arthritis detection, and facilitated the diagnosis of arthritis by identifying the features of angiogenesis in inflammatory joint diseases. Subsequently, a more in-depth study was conducted. iRGD peptide-functionalized echoliposomes (iELPs) encapsulating MTX and indocyanine green fluorescent probes were prepared using a thin-film hydration technology [107]. The prepared iELPs had a high affinity for αvβ3 integrins and good fluorescence tracking performance. During the progression of arthritis, inflammatory factors in the joint space stimulate endothelial cells and upregulate integrin αvβ314. In the mouse model of RA, inflammatory cell infiltration was significantly reduced by treatment with iELPs and ultrasound. In addition, the iELPs showed high affinity, good in vitro and targeted accumulation of acoustic response, and fluorescence tracking capability.

### Fluorescent probes for the detection of cysteine (Cys) as an RA biomarker

Biothiols such as glutathione, homocysteine (Hcy), and Cys play key roles as free radical scavengers in preventing oxidative damage and thus help to maintain redox homeostasis and regulate important cellular processes [108]. Cys, an amino acid, is a very important component of the cellular antioxidant system and is involved in numerous enzymatic reactions regulating vital cellular processes. Abnormal Cys concentrations can lead to slow growth, neurodegenerative diseases, loss of hair pigmentation, cardiovascular diseases, cancer, acquired immune deficiency syndrome, and liver damage [109, 110]. To determine the correlation between mitochondrial Cys concentration and arthritis during cellular oxidative stress, one should select a suitable method for accurately quantifying Cys concentration in situ.

To analyse the influence of mitochondrial Cys on arthritis, Wang et al. [110] designed a high-performance two-photon fluorescence probe, In-CYS, for sensitive monitoring of Cys fluctuations, and tested its performance. The acrylate group could selectively react with Cys (Michael addition reaction) to produce the corresponding thioether, yielding lactam byproduct and the dye In-OH with strong yellow fluorescence (Fig. 7c). The probe not only had high specificity to Cys, but also demonstrated strong sensitivity. In-CYS could be employed to specifically and sensitively measure fluctuations in in situ Cys levels through single- and two-photon fluorescence imaging. Moreover, the In-CYS method was used to successfully observe the reduction of Cys levels in the ankle joint of arthritic mice. Relevant studies have supported the formulation of arthritis treatment programs, clarified the correlation between mitochondrial oxidative stress and RA, and provided support for mechanistic research of such diseases.

### Fluorescent probes for the detection of other species [apoptosis-targeting peptide-1 (CApoPep-1) and Cystathione-1] as RA biomarkers

To study the key role of apoptosis in RA development, So et al. [111] proposed a fluorescent probe containing CApoPep-1 for imaging apoptosis. This probe can be used to spatially and temporally observe apoptosis in vitro and can also be isolated in vitro. The feasibility of visualizing and quantifying apoptosis using this probe was evaluated in a mouse model of collagen-induced arthritis (CIA), particularly for the early detection of in situ apoptotic responses after treatment. The CApoPep-1 signal was primarily colocalized with transferase dUTP nick end labeling signaling in well-defined cell populations of CIA mice. CApoPep-1 is useful for the noninvasive imaging of apoptosis in different inflammatory diseases. The recruitment and activation of cystathione-1 are markers of inflammatory vesicle activation, which highlights the importance of investigating the role of cystathione-1 in RA. Ren et al. [112] designed and synthesized two types of fluorescent probes, WEHD-HCy and YVAD-HCy, to detect their activity during inflammation. Cystathione-1, a direct marker of inflammatory vesicle activation, plays a vital role in the pathogenesis of various inflammatory diseases. Additionally, the above probes can be used to visualize the activation of endogenous cystein-1 by NOD-like receptor protein 3 inflammatory vesicles in living cells. These developed biosensors can be applied to the evaluation of different inflammatory diseases, such as *Salmonella* infection and acute RA models. The cystein-1-responsive fluorescent biosensors are efficient and convenient in situ tools for monitoring inflammatory vesicle activation and have potential applications in the clinical diagnosis of related diseases.

## Agents for imaging clinical RA progression

NIR fluorescence molecular imaging is a widespread method of intraoperative clinical imaging used to assist tumor removal while avoiding critical structures, and may also be used for the early diagnosis of diseases such as breast cancer and RA. Duan et al. [113] designed and synthesized a novel probe (IRDye-680RD-4-1BB mAb) to track 4-1BB-activated T cells. 4-1BB mAb was coupled to an IRDye-680RD NHS ester to enable efficient analysis based on a fluorescence signal. Immunofluorescence staining and random neighborhood envelope analysis of T-cell distribution showed that CD3 and 4-1BB were colocalized, indicating the predominant expression of 4-1BB. Moreover, 4-1BB was identified as a potent RA biomarker for detecting activated T-cells, and imaging enabled the noninvasive diagnosis of RA in vivo. In addition, this probe was used to determine 4-1BB-activated T-cells in mice with adjuvant-induced arthritis and diagnose RA. To improve the feasibility of optical imaging for early RA detection, a safe, cheap, and convenient drug delivery route would be ideal. In addition, to better apply fluorescent reagents in clinical studies, Bhatnagar et al. [114] developed potential molecular imaging tools for the oral administration of integrin-conjugated NIR imaging agents in an RA mouse model. Regardless of the administration mode, IRDye800CW was significantly absorbed in the inflamed joints of mice with collagen antibody-induced arthritis. The feasibility of imaging was demonstrated by 3D confocal imaging and theoretical calculations. The oral and subcutaneous delivery of NIR fluorescent molecular imaging agents (a potential self-administration route) can be used to detect joint inflammation at a sufficient level of contrast and with high potential to discriminate RA joints after clinically relevant and through oral administration. Despite the challenges associated with this approach, there is strong support for its clinical translation.

## Conclusions and outlooks

RA is a systemic autoimmune inflammatory disease that seriously harms human health and life quality. Patients with RA often experience prolonged pain, especially during sudden weather changes. Chronic pain can seriously damage health, cause irreversible damage to joints, and even lead to disability if left untreated. In addition, some drugs designed to alleviate or reduce the incidence of RA are not fully effective. Therefore, appropriate analytical methods for the early or postoperative diagnosis of RA should be developed to effectively hinder disease progression, ensure the normal life of patients, and avoid joint necrosis or disability.

In this review, we summarize the recent progress in the development of fluorescent probes for RA biomarker detection, classify these probes according to their biomarkers and properties, and describe the advantages of certain probe types. The detection and imaging of RA markers shed light on the pathogenesis of RA and the roles of these markers in RA development. We also believe that this review can help those interested in developing new probes for early RA diagnosis or treatment.

At present, most of the developed fluorescent probes focus on active small molecules (e.g., HClO, ONOO^−^, Cys), whereas those suitable for other substances such as metal ions and enzymes are scarce. Based on the current applications of fluorescent probes in RA research, we outline several possible directions for future research on fluorescent probes for RA diagnosis and treatment: (1) NIR or two-photon probes. NIR probes, particularly NIR-II and two-photon ones, are well suited for biological imaging because of the high permeability of tissues to low-energy light. The development of activatable NIR-I and NIR-II probes could provide more realistic information on active species (biomarkers) in deep animal tissues [83, 115]. In particular, NIR-II probes hold great promise for clinical applications in humans, which are currently scarce. (2) Fluorescent probes with ultrahigh sensitivity and specificity. Although these fluorescent probes have been used for specific imaging in living organisms, most of them are only used to image living cells, tissues, or mice that produce biomarkers when stimulated with chemicals such as LPS. Many probes with low LODs have been reported for the detection of HClO or ONOO^−^ but are still poorly suited for early-stage arthritis monitoring [58, 66, 87, 92]. In addition, the currently reported C=C bonds can be broken not only by HClO, but also by ONOO^−^ [62, 63, 116]. The internal environment of an organism is complex and variable, and the presence of certain species may interfere with the application of these probes. Thus, methods for the in situ real-time imaging of disease-specific markers and, hence, for the early diagnosis of RA, are highly sought after. (3) Fluorescent probes for the simultaneous detection of multiple markers. In general, disease development occurs through the participation of multiple species. The study of single species only may suffer from the presence of other interfering species or structural bias–inducing false positive signals. Therefore, probes capable of detecting multiple markers simultaneously are required. Gao et al. [117] designed pH- and β-galactosidase-responsive fluorescent probes to accurately track senescence and avoid other sources of interference. However, reports on the simultaneous detection of multiple RA markers using fluorescent probes are scarce. In the case of the simultaneous detection of multiple species, their synergistic effects can be clarified to shed light on the etiology and pathogenesis of RA and promote RA treatment. (4) Super-resolution fluorescent probes. The widespread application of super-resolution fluorescence microscopy helps to obtain more accurate information. Chai et al. [118] visualized the cellular-level distribution of enzymes at an unprecedented nanoscale using a super-resolution probe. The combination of super-resolution microscopy with fluorescent probes allows a comprehensive understanding of the relevant physiological or pathological processes involved. (5) Development of multimodal fluorescent probes. Single techniques often have certain limitations, while the combination of multiple techniques can improve detection accuracy. For example, developments in photoacoustic imaging, photothermal therapy, and other technologies have facilitated favorable therapeutic integration. Li et al. [119] developed a multimodal activatable imaging probe for the in vivo fluorescent photoacoustic and radioactive signal imaging of biomarkers related to prostate cancer diagnosis and prognosis (i.e., liver proteases and prostate-specific membrane antigens) and thus paved the way for the early diagnosis of prostate cancer. However, the use of such probes in RA studies has rarely been reported. We believe that the simultaneous detection of multiple markers and the combined use of multiple techniques could facilitate the early detection of RA.

Overall, this paper comprehensively summarizes progress in the design of fluorescent probes for RA therapy and their application in bioimaging. In the near future, fluorescence imaging is expected to find widespread use in the early diagnosis and treatment of RA while providing more assistance in biomedical and clinical fields.

## Data Availability

Not applicable.

## Abbreviations

AIE: Aggregation-induced emission
CApoPep-1: Apoptosis-targeting peptide-1
Cys: Cysteine
ESIPT: Excited state intramolecular proton transfer
FRET: Fluorescence energy resonance transfer
HClO: Hypochlorous acid
HNO: Nitroxyl
ICT: Intramolecular charge transfer
LPS: Lipopolysaccharide
MPO: Myeloperoxidase
MTX: Methotrexate
NIR: Near-infrared
·OH: Hydroxyl radical
ONOO^−^: Peroxynitrite
RA: Rheumatoid arthritis
RONS: Reactive oxygen and nitrogen species
UCNPs: Upconversion nanoparticles
MRI: Magnetic resonance imaging
LOD: Limit of detection
BODIPY: 10-[4-(2,5-dioxo-2,5-dihydro-1 H-pyrrol-1-yl)phenyl]-5,5-
DSNP: Lanthanide-doped down-shifting nanoparticle
Hcy: Homocysteine
